# Assessing Learner Engagement and the Impact on Academic Performance within a Virtual Learning Environment

**DOI:** 10.3390/pharmacy11010036

**Published:** 2023-02-15

**Authors:** Suzanne Galal, Deepti Vyas, Martha Ndung’u, Guangyu Wu, Mason Webber

**Affiliations:** 1 Thomas J. Long School of Pharmacy, University of the Pacific, Stockton, CA 95211, USA; 2 Fu Foundation School of Engineering and Applied Science, Columbia University, New York, NY 10027, USA

**Keywords:** self-regulated learning, time-management, self-efficacy, metacognition, test anxiety

## Abstract

Background: The objective of this pilot study was to examine student engagement with weekly self-paced learning materials in a virtual therapeutics course, and how sub-factors in the Motivated Strategies for Learning Questionnaire (MSLQ) may have influenced academic performance. Methods: Students within a diabetes module of a therapeutics course were provided with weekly asynchronous optional self-directed learning activities. Student submissions, on-time rates, self-reported weekly study time, and exam performance were collected. Students completed the MSLQ at the completion of the study. Data was evaluated using various correlation analyses to determine the predictive ability of the MSLQ and its 5 subscales. Results: In total, 173 students completed the study. Students’ self-efficacy score on the MSLQ subscale and case submission on-time rate have the strongest positive correlation with the exam score, while the test anxiety as reported on the MSLQ test anxiety subscale had the strongest negative correlation with the exam score. Conclusions: Study results proved the MSLQ to be an effective predictive tool in students’ self-regulation skills. Results can be used to develop intentional interventions aimed at improving self-regulation skills while providing opportunities to enhance student learning.

## 1. Introduction

Pharmacy students are faced with intense course loads, experiential learning requirements, extra and co-curricular activities, all of which require self-regulation skills for academic success. Self-regulated learning refers to the ability to understand and control a learning environment [1,2]. This is done by setting goals and selecting strategies to help achieve those goals while implementing strategies to monitor progress [1,2]. Self-regulation is likely to be an important predictor of students’ use of instructional strategies and materials, which, in turn, impacts learning [3]. The virtual learning environment creates additional challenges for those students who lack skills in self-regulation due to difficulties in managing their learning process, which can result in high dropout and low retention rates [4,5]. Previous studies support the theory that self-regulated learners are more successful in self-paced open and distance learning, while students with low self-regulating skills struggle with courses that have increased learner-control, especially within the virtual learning environment [6,7,8,9].

The Motivated Strategies for Learning Questionnaire (MSLQ) was created to assess students’ motivational orientations and their use of various learning strategies [10]. It contains 5 sub-factors; self-efficacy (SE), intrinsic value (IV), cognitive strategies use (CS), test anxiety (TA), and self-regulation (SR). The MSLQ is a self-reported questionnaire that reflects students’ learning and motivation in a specific course and responses may vary as a function of the task, situation, or school context [11]. It is one of the most widely used instruments designed to measure student motivations for learning and has been used in various educational settings and disciplines [11,12,13,14,15]. Within health professions, components of the MSLQ have proven to be reliable in predicting academic achievement [16,17,18]. Within pharmacy education, there is limited research determining how the MSLQ and its sub-factors relate to course engagement.

The objective of this study is to examine the relationships between student engagement with weekly self-paced learning materials in a virtual therapeutics course with the sub-factors in the MSLQ and the impact on academic performance.

## 2. Materials and Methods

**Figure 1:**
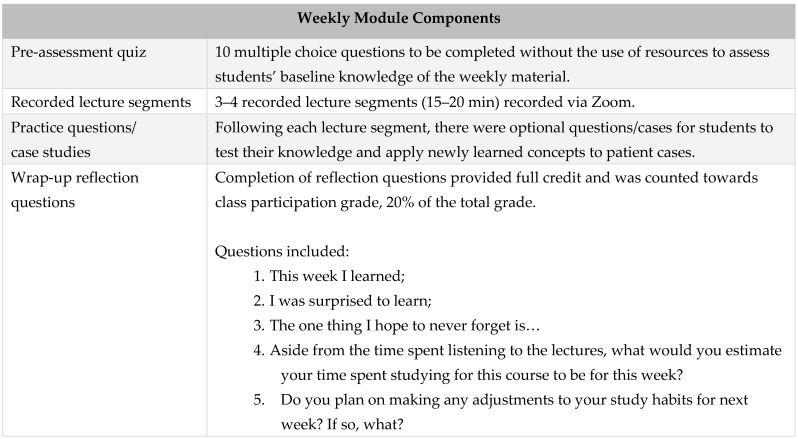
Weekly Module Components.

This study used a retrospective observational study design examining data from a required 2 unit endocrine integrated science course in the PharmD curriculum. This study took place during the diabetes module over a 6 week timeframe. The course used a self-paced, flexible modular design delivered via an online learning management system, Canvas. Each weekly module consisted of four major components: pre-assessment quiz, recorded lecture segments, practice questions/cases, and a wrap-up reflection. (Figure 1) 

Each module was posted 1 week prior to the synchronous discussion in which the instructor walked the students through the weekly case studies. This discussion session was optional for students. There was no grade assigned for the completion of the activities besides the class participation points provided to those who completed the wrap-up reflection questions at the end of the module. At the completion of the 6 week diabetes module, students were formally assessed on their knowledge and application of the material. This assessment contained 45 multiple choice case-based questions and counted for 30% of their total grade.

As part of the course introduction, the instructor briefly (10 min) discussed effective evidence-based study habits and introduced the concepts of self-regulated learning. At the end of the module, students completed the MSLQ [10]. This is a 44 item survey that uses 5 subscales: self-efficacy (SE), intrinsic value (IV), cognitive strategies use (CS), test anxiety (TA), and self-regulation (SR). Students rate themselves on a 7-point Likert scale, from 1 (not at all true of me) to 7 (very true of me). Scores for the individual subscales are computed by taking the mean of the items within that subscale. The MSLQ does not use norms but rather asks student to respond to questions regarding their learning and motivation in that specific course [11].

The student data was retrieved from Canvas and analyzed to determine student engagement with course material including; case submission, case submission on time rate (submitted within 1 week from the posted weekly module date), case attempts rate, and the self-reported weekly study time for this course. Negative standard deviation of study time was calculated from the average study time across the study as a proxy to determine students’ consistency with their study habits, with a larger standard deviation indicating more variation in reported weekly study time. Data analysis was performed to determine correlations between student engagement with course materials and the MSLQ and its sub-factor scores, as well as with academic performance, as it relates to the total score on the exam administered at the end of the diabetes module and study duration.

Data analysis was performed to assess correlation and correlation significance between study habit data, exam score, and MSLQ results. Frequencies are measured by demographic items. Pearson’s r was applied to measure the strength of the correlation. Statistical significance was set at *p* < 0.05. Statistical analysis is performed with the Scipy Stats module in Python, and Excel 2013 was used for data collection, storage, and analysis [19,20].

## 3. Results

There was a total of 173 students included in the analysis. We observe a statistically significant positive correlation between students’ practice case submission rate and their exam score (*p* < 0.05). Positive correlation also exists for students who submitted the practice cases on time and those who made multiple attempts. Submission rate and on-time rate have a significant positive correlation, suggesting that students who submit more cases throughout the semester also tend to submit on time each week (Table 1).

We then looked at the MSLQ scores to determine which subset of learning strategies might serve as the biggest influences on academic performance. Of the 5 different subscales, students self-efficacy, self-regulation and intrinsic value skills had the strongest positive correlation with students’ exam performance (Table 2).

To understand how MSLQ score may affect academic performance through different study habits, we further analyzed the correlation between the MSLQ and study habits within two subsets of the subject population. For students who score equal or higher than the median score in the final exam, we observe strong positive correlations between case submission rate and students’ self-efficacy, self-regulation, and intrinsic value skills (Table 3).

For the students who score lower than the median score, a different set of MSLQ skills are at play. While intrinsic value score still has a significant positive correlation with case submission rate, self-regulation now positively correlates with the extra attempts students use and their median study time (Table 4).

## 4. Discussion

It is difficult to know how and to what extent students engage with course materials, especially with a large class size. Beyond performance outcomes, there is limited information regarding how specific instructional materials are used and if they are beneficial to the learner or contribute to their course performance. The exploration of the learner’s study habits, as well as their motivations for learning in a specific course, may allow for a better understanding of how learner engagement with the course can be enhanced [17].

This study examined the study habits of students as it relates to their activity with weekly self-paced learning modules within a therapeutics course. Results showed that those students who completed the weekly materials, those with multiple attempts, and those who completed the activities multiple times had a statistically significant positive correlation with their exam performance. This finding was encouraging as it indicates the module activities were in fact beneficial to the learner. The strongest positive correlation with exam performance was seen in students who consistently submitted the materials each week by the recommended due date. This finding might be explained by the “spacing effect” in which spacing study out into multiple sessions promotes greater long-term learning versus massing (cramming) study into one study session prior to the exam. While a number of studies have shown that the spacing effect is a significant positive predictor of academic success, there are some studies that have not [21,22,23,24]. This may be due to the specific type of content or skill being learned and the variability in the measurement of students’ study habits. 

We then looked at the MSLQ scores to determine which subset of learning strategies might serve as the biggest influences on academic performance. Of the 5 different subscales, students self-efficacy, self-regulation and intrinsic value skills had the strongest correlation with students’ exam performance. This is consistent with previous findings that show students’ self-reported measure of self-efficacy and self-regulation skills is a significant predictor of academic performance [25,26,27,28,29].

Selecting particular aptitude or event measures to identify what influences self-regulated learning is critical in gaining an accurate account of these skills [30,31]. While we used a combination of aptitude and event measures to help us identify learning behaviors and the influences, limitations should be noted. This study used a correlational design which does not allow for causal inferences about the observed correlations. Experimental research is needed to determine if specific interventions can impact students’ academic performance as it relates to their motivation for learning and self-regulation skills. There are also limitations with the measures used in this study. Mixed results have been reported on how well the subscales of the MSLQ can be linked to course activity measures or trace data collected [30,31,32,33,34]. This may be due to the dynamic and multidimensional process involved in learning strategies and behaviors. Additionally, the tool is designed to be course specific and students answer the questions as it relates to their specific course. This may limit the generalizability of our findings across activities in other courses or medical education populations [11]. This study did not collect demographic information, however, future research should determine how social determinants of learning may affect the validity and utility of the tool. Furthermore, the MSLQ has not been widely studied in diverse populations [35]. A comparative analysis and the determining factors within a diverse population, including students from other countries, should be carried out. Despite the limitations, the measures and findings of this study allowed for a better understanding of learners’ engagement and behaviors within this population and may provide the grounds for future research in this area.

Future studies can explore best practices for students based on using the MSLQ results as a tool to better understand their own learning behaviors and self-regulation skills strengths and weaknesses. This is important to know, as self-regulated learning skills can be learned and developed [7,36,37]. This information can inform both students and instructors on what the best learning environment is to achieve success. For example, this information can be used as a course orientation tool for students to use in making decisions on how to engage with the course material that best suits their own learning behaviors and motivations. Instructors can use this information to develop more structure, impose deadlines and have continuous prompting throughout the semester, which has been shown to lead to improvements in self-regulation, learning, and retention [38]. A beneficial exercise for those who need improvement with self-regulation may consist of teaching and guiding students on how to goal set and manage time with course content and assessments. Further exploration should be done into the use of technology, such as open student models or virtual coaches linked to existing learning management systems, in order monitor study habits and self-regulation skills, and to provide real-time feedback to students [39,40].

## 5. Conclusions

The study results provided insight on learner engagement and how the MSLQ may be an effective predictive tool in students’ study habits and self-regulation skills. The results can be used to develop intentional interventions aimed at improving self-regulation skills while providing opportunities to enhance student learning. Future research should explore the effectiveness of specific interventions in improving self-regulation skills. 

## Figures and Tables

**Table 1:** Correlations between students’ exam scores and study habits.

Measure	Mean	SD	Exam Score	Case Submission	On-Time Submission	Extra Attempts	Median Study Time
Exam Score	0.85	0.09					
Case Submission	0.65	0.35	0.17 *				
On-time Submission	0.19	0.3	0.28 ***	0.37 ***			
Extra Attempts	0.05	0.13	0.17 *	0.31 ***	0.4 ***		
Median Study Time	2.48	1.72	0.04	0.03	−0.12	0.04	
SD of Study Time	1.64	1.69	−0.01	0.13	−0.15	0.01	0.45 ***

* = *p* < 0.05; *** = *p* < 0.001. SD = Standard Deviation.

**Table 2:** Correlations between students’ exam scores and post MSLQ sub-factor scores.

Measure	Mean	SD	Exam Score	MSLQ-CS	MSLQ-IV	MSLQ-SE	MSLQ-SR
Exam Score	0.85	0.09					
MSLQ-CS	5.09	0.64	0.10				
MSLQ-IV	5.96	0.67	0.21 **	0.63 ***			
MSLQ-SE	5.10	1.04	0.35 ***	0.58 ***	0.56 ***		
MSLQ-SR	4.93	0.75	0.23 **	0.62 ***	0.58 ***	0.62 ***	
MSLQ-TA	4.89	1.45	−0.14	−0.01	−0.01	−0.25 **	−0.31 ***

** = *p* < 0.01; *** = *p* < 0.001. SD = Standard Deviation; MSLQ = Motivated Strategies for Learning Questionnaire; CS = Cognitive Strategy; IV = Intrinsic Value; SE = Self Efficacy; SR = Self-Regulation; TA = Test Anxiety.

**Table 3:** Correlations between study habits and MSLQ sub-factor scores in students scoring greater than the median on the exam.

Measure	Mean	SD	Case Submissions	Submission on Time	Extra Attempts	Median Study Time	SD of Study Time	MSLQ-CS	MSLQ-IV	MSLQ-SE	MSLQ-SR
Case Submissions	0.65	0.35									
Submission on time	0.19	0.3	0.4 ***								
Extra Attempts	0.05	0.13	0.32 **	0.42 ***							
Median study time	2.48	1.72	−0.03	−0.18	0.06						
SD of study time	1.64	1.69	0.17	−0.19	0.04	0.4 ***					
MSLQ-CS	5.09	0.64	0.2	−0.03	0	0	0.1				
MSLQ-IV	5.96	0.67	0.24 *	0.02	0.02	−0.06	0.09	0.63 ***			
MSLQ-SE	5.1	1.04	0.27 **	0.19	0.13	−0.04	−0.08	0.56 ***	0.54 ***		
MSLQ-SR	4.93	0.75	0.29 **	0.1	−0.07	−0.05	−0.02	0.62 ***	0.59 ***	0.52 ***	
MSLQ-TA	4.89	1.45	0.06	−0.05	0.12	0.05	0.21 *	−0.13	−0.15	−0.39 ***	−0.46 ***

* = *p* < 0.05; ** = *p* < 0.01; *** = *p* < 0.001. SD = Standard Deviation; MSLQ = Motivated Strategies for Learning Questionnaire; CS = Cognitive Strategy; IV = Intrinsic Value; SE = Self Efficacy; SR = Self-Regulation; TA = Test Anxiety.

**Table 4:** Correlations between study habits and MSLQ sub-factor scores in students scoring less than the median on the exam.

Measure	Mean	SD	Case Submissions	Submission on Time	Extra Attempts	Median Study Time	SD of Study Time	MSLQ-CS	MSLQ-IV	MSLQ-SE	MSLQ-SR
Case Submissions	0.65	0.35									
Submission on time	0.19	0.3	0.27 *								
Extra Attempts	0.05	0.13	0.28 *	0.26 *							
Median study time	2.48	1.72	0.06	−0.07	−0.03						
SD of study time	1.64	1.69	0.07	−0.07	−0.09	0.53 ***					
MSLQ-CS	5.09	0.64	0.02	0.12	0.18	0.14	0.11				
MSLQ-IV	5.96	0.67	0.24 *	0.2	0.16	0.11	0.16	0.63 ***			
MSLQ-SE	5.1	1.04	0.06	0.28 *	0.17	0.21	0	0.61 ***	0.57 ***		
MSLQ-SR	4.93	0.75	−0.02	0.15	0.22 *	0.26 *	0.13	0.64 ***	0.55 ***	0.7 ***	
MSLQ-TA	4.89	1.45	0.1	−0.17	−0.09	0.05	0.01	0.12	0.14	−0.1	−0.11

* = *p* < 0.05; *** = *p* < 0.001. SD = Standard Deviation; MSLQ = Motivated Strategies for Learning Questionnaire; CS = Cognitive Strategy; IV = Intrinsic Value; SE = Self Efficacy; SR = Self-Regulation; TA = Test Anxiety.

## Data Availability

The data presented in this study are available on request from the corresponding author.
